# Changes in Hypertension-Related Knowledge and Behavior and Their Associations with Socioeconomic Status among Recently Urbanized Residents in China: 2013–2016

**DOI:** 10.3390/ijerph15081701

**Published:** 2018-08-09

**Authors:** Wenjie Zhang, Hongdao Meng, Shujuan Yang, Honglin Luo, Danping Liu

**Affiliations:** 1 Department of Health and Social Behavior, School of Public Health, Sichuan University, Chengdu 610041, China; howwenj@163.com (W.Z.); rekiny@126.com (S.Y.); 2 School of Aging Studies, College of Behavioral &Community Sciences, University of South Florida, Tampa, FL 33620, USA; meng@usf.edu; 3 He Zuo Community Health Service Center in Chengdu Hi-Techzone, Chengdu 610041, China; lhl008@163.com

**Keywords:** hypertension, health knowledge, health behavior, urbanization, recently urbanized residents, socioeconomic status, China

## Abstract

The rapid urbanization in China has brought with it some health benefits, but it also brought about a negative influence on the lifestyle of residents. We conducted this study to assess the change in hypertension-related knowledge and behavior from 2013 to 2016 among recently urbanized residents and determine their association with socioeconomic status (SES). This research used data from two cross-sectional studies conducted in Hezuo community in Chengdu, Sichuan province of China. A total of 2268 and 2601 individuals, respectively, participated and completed standard questionnaires. According to the results, the median (IQR) scores of health knowledge was 1 (0,3) and 3 (1,5), respectively, (*p* < 0.001) and the median (IQR) scores of health behavior was 6 (5,6) and 5 (5,6), respectively, (*p* < 0.001) in 2013 and 2016. The rate of sufficient knowledge increased from 8.8% to 18.1% (*p* < 0.001), while the rate of correct behavior decreased from 54.5% to 45.5% (*p* < 0.001) in three years. Logistic regression analysis showed that higher education was associated with sufficient hypertension-related knowledge (*p* < 0.05), and those with higher education, unemployment, and retirement were more likely to have sufficient behavior (*p* < 0.05). The impact of SES on knowledge was stable between 2013 and 2016. The behavior difference between the middle school educated and the illiterate increased from 2013 to 2016 (*p* < 0.05), and the behavior difference between the unemployed and manual workers decreased from 2013 to 2016 (*p* < 0.05). Our results revealed that hypertension-related knowledge improved with no corresponding improvement in self-reported behavior among recently urbanized residents from 2013 to 2016. Organizational strategy should be implemented to improve health education on knowledge, and what is more, translate knowledge into behavior. All these measures should be given more attention to the lower educated and manual workers among recently urbanized residents to eliminate the SES disparity.

## 1. Introduction

It is estimated that 80% of deaths and 70% of disability-adjusted life-years lost are caused by non-communicable diseases (NCDs) in China [1]. Hypertension is the most common NCD and its main complication, cardiovascular diseases (CVD), has a high mortality and morbidity [1,2]. Based on large-scale population data, the age-standardized and sex-standardized prevalence of hypertension has already reached 37.2% in China, and only 50% of hypertension patients take medication and only 20% are under effective control overall [3]. As a result, uncontrolled hypertension accounted for about one-third of deaths from CVD [4].

Hypertension prevention and control is one of the top public health priorities in China. In 2009, the Chinese government implemented the Essential Public Health Services (EPHS), a national health service program promoting the management of NCDS represented by hypertension and diabetes in primary care [5]. The hypertension service for EPHS focuses on follow-up and management for hypertension patients and developing primary prevention of related education on knowledge and behavior for all residents [6]. 

Many behaviors have been proved to be modifiable risk factors of hypertension such as smoking, alcohol use, lack of exercise, excessive salt intake, excessive pickled food intake, and so on [7,8,9,10], sufficient hypertension-related knowledge and correct behavior form the basis for hypertension prevention and control [11,12]. Socioeconomic status (SES), which is consisted of factors such as education, occupation, and income, is considered to be the determinant of health-related knowledge and behavior, individuals with low SES more often have poor knowledge and unhealthy behavior [13]. Some studies have demonstrated the influence of SES on hypertension-related knowledge [14,15]. Xia Li et al. revealed that hypertension-related knowledge of the illiterate was always lower than those with primary or higher education in rural areas of China, whether in hypertensive or nonhypertensive individuals [14]. Fazel et al. showed that low income and low level of education were associated with poor hypertension-related knowledge among adults in southern Iran [15]. Other studies also revealed the relationship between SES and hypertension-related behavior [16,17,18,19]. Smokers with lower education showed less willingness to quit and fewer quit attempts and reported a younger age in their smoking history [16]. A study revealed that sodium intake was associated with income levels among Japanese workers, and people with low incomes are less likely to use low-salt foods or follow salt restrictions [17]. Both urban and rural residents are less likely to partake in excessive drinking compared with high education and low incomes in China [18]. Even in patients with confirmed hypertension, lower education and incomes were also less likely to report involvement in blood pressure control [19]. However, there is no study that has comprehensively explored the effects of SES on hypertension-related knowledge and behavior among recently urbanized residents.

China has experienced rapid economic development and urbanization since the late 1970s, with a rapid increase in city size and population [20]. The urban population exceeded the rural population for the first time in 2011, and an estimated 1 billion residents will live in urban areas by 2030 [21]. Benefiting from the enormous political and economic power provided by the cities, the recently urbanized residents are experiencing a great transformation in their lives because of the great advantage of cities in terms of the high degree of information, convenient health services, rich education resources, and other social welfare services [22]. One of the beneficial consequences of this transformation for the recently urbanized residents is that more resources are available to acquire information.

However, the negative consequence of this transformation is increased behavior associated with hypertension risk factors. For example, the level of physical activities in work and family for adults has fallen by nearly half between 1991–2011, the increasing sedentary behavior for residents is negatively correlated with living in more urbanized areas which accompanies the increasing of car dependency and screen time on mobile phones and computers [23,24,25]. Results from the national China Health and Nutrition Survey showed that the energy that urban residents get from processed food is just about the double of rural residents, suggesting excessive salt intake [26]. 

The purpose of this study is to explore the changes of hypertension-related knowledge and behavior situation among recently urbanized residents in three years from 2013 to 2016 and its association with SES in China. Under the fast growth economy and sustained urbanization of China, it has significant practical implications to choose the recently urbanized residents as subjects, and this is the first study to explore hypertension-related knowledge and behavior among recently urbanized residents.

## 2. Materials and Methods

### 2.1. Setting and Participants

This research used data from two cross-sectional studies conducted in Hezuo community in Chengdu, Sichuan province in 2013 and 2016. The Hezuo community is located in the Chengdu High-tech Development Zone which started to change from rural to urban in 2006, the majority of its residents are still in the rural urbanization process. The gross domestic product (GDP) of high-tech development zones was 18.22 billion yuan in 2006, and it increased to 103.97 billion yuan in 2013, and 143.65 billion yuan in 2016 [27,28,29]. Residents aged ≥18 years and lived in the Hezuo community for no less than 6 months qualified for this study. A multistage stratified random sampling survey was used to acquire the sample. First, we randomly selected six of nine community neighborhood committees in the Hezuo district. Next, five building units were randomly selected from each selected community neighborhood committee. Then, we used systematic random sampling to choose 90 and 100 representative families in the chosen building units, respectively, in 2013 and 2016. The sample of this study was based on family unit and each family randomly chose one resident as the respondent. On the basis of informed consent, each participant took a face-to-face interview guided by an appropriately-trained investigator and completed the questionnaire anonymously. There were 2268 and 2601 residents that participated in the survey in 2013 and 2016, respectively (the effective response rate was 84.0% and 86.7%). The Public Health School of Sichuan University Institutional Review Board approved the protocol (Project identification code: H160311).

### 2.2. Measures

The questionnaire was designed according to the community health assessment standardized questionnaires in China [30]. 

#### 2.2.1. SES

This study selected three most representative SES measures: education, occupation, and monthly income. Education was defined as the highest level of qualification that has been completed and was divided into five levels: illiterate, elementary school, middle school, senior school, or technical secondary school, junior college, and above. Occupation was grouped into four categories, which were manual work, nonmanual work, unemployment, and retirement. Monthly income was classified as less than 1000CNY, 1000–2499CNY, 2500–3999CNY, and more than 4000CNY.

#### 2.2.2. Other Sociodemographic Characteristics

There are other sociodemographic characteristics contained in study including gender, age, marital status, and self-reported hypertension.

#### 2.2.3. Hypertension Related Knowledge

The instrument of hypertension related knowledge contains eight items: one question about the recommended daily intake of salt for adults, one question about whether hypertension patients should take medicine for life, and another six questions about risk factors of hypertension. Each correct answer gains 1 point and the wrong answer gains 0 points, the total score of hypertension related knowledge ranges from 0 to 8 and sufficient hypertension related knowledge was defined as the correct rate higher than 80%.

#### 2.2.4. Hypertension Related Behavior

The instrument of hypertension related behavior contains seven items: smoking (regularly, quit, or never), alcohol use (regularly, occasionally, or never), salt intake (low or high), smoked or pickled food intake (low or high), exercise (regularly, occasionally, or never), weight control (regularly, occasionally, or never), and measure blood pressure (regularly, occasionally, or never). Healthy behavior included quit or never smoking, occasionally or never drinking, low salt intake, low smoked or pickled food intake, exercise regularly, weight control, and measure blood pressure regularly. Each healthy behavior gains 1 point and the unhealthy behavior gains 0 points, the total score of hypertension related behavior ranges from 0 to 7 and sufficient hypertension related behavior was defined as a healthy behavior formation rate higher than 80%.

### 2.3. Statistical Analysis

First, we used descriptive statistics to present the SES and other demographic characteristics of participants, including education, occupation, monthly income, gender, age, marital status, and self-reported hypertension. Second, both the overall score level of hypertension related knowledge and behavior and the correct rate of each item were presented. We used the number and percentages to describe categorical variables and the median and interquartile range to describe non-normal continuous variables. Changes in each item was analyzed by logistic regression and presented by the odds ratios (ORs) and 95% confidence intervals (CIs), changes in the overall score was tested by the Wilcoxon rank sum test. Next, we used logistic regression analysis to estimate the relationship between SES and sufficient hypertension related knowledge and behavior in two models. In model 1, we tested the main effects of education, occupation, monthly income, and other confounding influencing factors. In the model 2, the interaction effect between survey years and SES was analyzed. *p* < 0.05 was considered statistically significant. Data from this study was recorded with Epidata 3.1 (The Epidata Association, Odense, Denmark) and statistical analyzed by SPSS 23.0 (SPSS Inc., Chicago, IL, USA).

## 3. Results

Table 1 shows the demographic characteristics of 2268 respondents in 2013 and 2601 respondents in 2016. The average age of respondents was 46.85 ± 17.08 years in 2013 and 46.85 ± 17.13 years in 2016. About half of respondents were female (52.8% and 53.4%, respectively). Most of respondents were married (82.2% and 83.5%, respectively). Those with a degree below middle school were 39.3% and 31.9%, respectively, and those with a junior college and above degree were 10.4% and 20.5%, respectively. Most of respondents were unemployed (41.9%) in 2013 and engaged in nonmanual work (39.5%) in 2016. The monthly income level of most respondents was concentrated in 1000–2499 CNY (63.4% and 58.5%, respectively). 10.9% and 11.5% of respondents self-reported suffering from hypertension in 2013 and 2016. Differences are statistically significant in education, occupation, and monthly income between 2013 and 2016.

The correct rate and changes of hypertension related knowledge and behavior among respondents are presented in Table 2. In the field of knowledge, the lowest correct question was “people should consume no more than 6 g of salt per day” which less than 10% of respondents answered correctly in 2013 (4.7%) and 2016 (7.5%). A significant increase in the correct rate of hypertension related knowledge was found for the risk factors including obesity, excessive salt intake, mental stress, smoking, and lack of exercise (*p* < 0.001) from 2013 to 2016. In the field of health behavior, the highest correct behavior was weight control both in 2013 (96.3%) and 2016 (93.6%). The lowest correct behavior was exercise regularly (53.4%) in 2013 and take blood pressure regularly (27.0%) in 2016. A significant increase (*p* < 0.001) in correct rate of hypertension related behavior was found in eating little smoked or pickled food and exercising regularly, while a significant decrease (*p* < 0.001) was found in drinking, weight control, and taking blood pressure regularly from 2013 to 2016.

Table 3 describes the overall score of hypertension related knowledge and behavior. The median (IQR) scores of health knowledge was 1 (0,3) and 3 (1,5) in 2013 and 2016, respectively, with statistical significance (*p* < 0.001). The median (IQR) scores of healthy behavior was 6 (5,6) and 5 (5,6) in 2013 and 2016, respectively, with statistical significance (*p* < 0.001).

**Figure 1:**
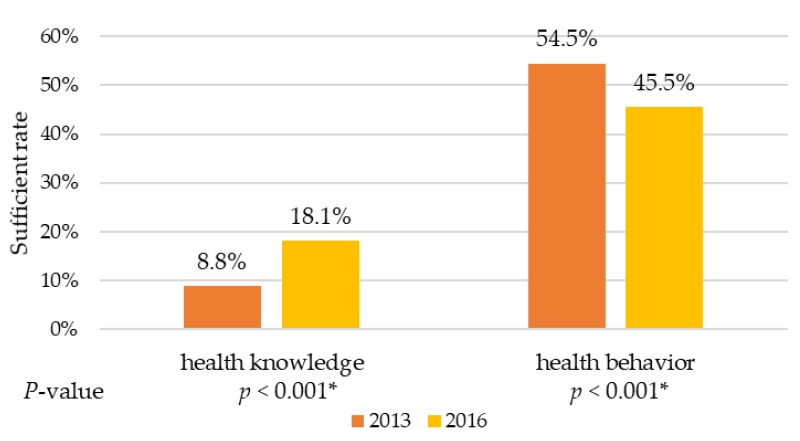
Overall sufficient rate of hypertension related knowledge and behavior, adjusted for age, gender, marital status, education, occupation, monthly income, and self-reported hypertension. * Statistically significant differences were detected both in knowledge (OR = 2.02, 95% CI: 1.67–2.47) and behavior (OR = 0.60, 95% CI: 0.53–0.69).

Figure 1 shows the changes of sufficient rate of hypertension related knowledge and behavior. The sufficient rate of knowledge increased from 2013 (8.8%) to 2016 (18.1%) with statistical significance (*p* < 0.001), and the sufficient rate of behavior decreased from 2013 (54.5%) to 2016 (45.5%) with statistical significance (*p* < 0.001).

Table 4 shows the association between SES and hypertension related knowledge and behavior. In the field of knowledge, respondents with higher education were more likely to have sufficient knowledge after considering gender, age, marital status, and self-reported hypertension (*p* < 0.001). And respondents who self-reported suffering from hypertension were more likely to have sufficient knowledge (*p* < 0.001). As shown in model 2, the interaction effects between SES and knowledge were not significant, suggesting that the impact of SES on knowledge was stable between 2013 and 2016. In the field of behavior, the higher educated, unemployed, and retired individuals were more likely to have sufficient behavior after considering gender, age, marital status and, self-reported hypertension (*p* < 0.001). Respondents who self-reported suffering from hypertension, female, older age, divorced, or widowed were more likely to have sufficient behavior (*p* < 0.001). At the same time, respondents with sufficient knowledge were more likely to have sufficient behavior (*p* < 0.001). As shown in model 2, the behavior difference between the middle school and the illiterate increased from 2013 to 2016 (*p* < 0.05). The behavior difference between the unemployed and manual workers decreased from 2013 to 2016 (*p* < 0.05).

## 4. Discussion

It was demonstrated that an improvement in knowledge and changes in behavior can largely prevent and control hypertension [31]. China implemented the community-based comprehensive prevention and control for hypertension involved in EPHS in urban areas which focuses on risk factor control [32]. As a major means of controlling risk factors, health education activities include effective communication between general practitioners and residents, distribution of knowledge manuals, health education lectures provided by CHCs, and so on. Along with the recent development of community health service, the recently urbanized residents can use convenient and economical hypertension services from CHCs, e.g., many CHCs provide free blood pressure measurement services to residents in China [33].

The level of hypertension related knowledge among recently urbanized residents has improved in three years (*p* < 0.001). With the development of urbanization and the information technology revolution mass media has transmitted and shared various health information that allow people to live in a health related knowledge-rich society [34]. Excellent medical service resources concentrated in cities has increased the availability of high quality health related knowledge [20]. However, the sufficient rate of hypertension related knowledge was only 18.8% and the correct rate of some items was still below 50% in 2016. Only 37.8% (2016) respondents were aware that mental stress was a risk factor for hypertension. As a response of the human body to the stimulation of psychological and physiological factors in the environment, negative mental stress can significantly increase hypertension risk [35]. And for recently urbanized residents, adapting to the pressures of strange societies or different lifestyles may lead to or aggravate their mental health problems [36]. Only 37.8% (2016) respondents were aware of smoking as a risk factor for hypertension. Smoking places people at serious risk of atherosclerosis disease, the recently urbanized residents probably faced a greater smoking risk after transforming into a higher income and higher consumption environment, it is also influenced by their life stress and mental problems [37,38]. There were only 35.4% (2016) respondents who recognized adequately that regular activity contributes to energy consumption and the stability of cardiovascular system [39]. Only 42.3% (2016) of respondents realized that people with hypertension should take drugs for life once they are diagnosed to influence blood pressure control [40]. Despite 62.1% (2016) of respondents recognizing the dangers of excessive salt intake, only 7.5% (2016) knew that adults should consume no more than 6 g of salt per day; this situation has not changed in three years.

The level of hypertension related behavior among recently urbanized residents has decreased in three years, the overall health behavior formation rate in 2016 (45.5%) is lower than in 2013 (54.5%) (*p* < 0.001). Less respondents engaged in appropriate alcohol use, weight control, and blood pressure measuring regularly in 2016 than in 2013 (*p* < 0.001). Under the process of urbanization, alcohol use in China is increasing rapidly, and the average annual alcohol consumption of Chinese people over the age 15 has increased from 0.4 L in 1952 to 4.9 L in 2009 [41]. One reason for the change in drinking behavior in China is that it is mainly used to promote social intercourse or business meetings which are more common in work and life in the modern city [26]. The 2013 China Chronic Disease and Risk Factor Surveillance study revealed the drinking rate of urban residents (39.1%) is higher than that of rural residents (35.4%) [42]. Weight control of residents is also constrained by more factors accompanies with urbanization for several reasons. Living in urban areas can increase the odds of doing nonmanual work and using vehicles for transportation which reduce energy consumption [43,44]. Mobile phones, television, and the Internet fill the leisure time of urban residents and increase their sedentary behavior [45]. In addition, the measurements of blood measure decreased strikingly in three years, the possible reasons for this situation should be further explored in the later research.

Overall, hypertension related knowledge has improved for the recently urbanized residents, while the related behavior gets worse from 2013 to 2016. This result verifies that the effect of urbanization on residents is double-edged. In addition, this result is consistent with the theory that it is the ability to respond to knowledge, rather than the knowledge itself, that promotes changing behavior [13]. Health knowledge forms the basis for behavior, but behavior can also be influenced by many factors, such as health belief, self-efficacy, and the individual’s social environment [46,47,48].

There were 39.3% and 31.8% of respondents respectively in 2013 and 2016 with a lower education than middle school, which showed lower degree of education among recently urbanized residents. After adjusting for potential confounding factors, the higher education respondents were found more likely to have the sufficient hypertension related knowledge and behavior which is consistent with previous studies [49,50]. Education develops the ability of individuals to access, evaluate, and use information, enabling them to seek valuable health knowledge [51]. Well-educated people are easier to adopt health behavior based on their conscious of prevention [52,53]. Education also brings resources to improve health behavior including a better social support and a feeling of being in control of one’s life [54]. Poor education not only has an effect on smoking, alcohol use, salt intake, and other risk behaviors, but also has difficulty in overcoming problems existed in the process of changing behavior, for example, Naik et al. found that poor education contributed to continued smoking and continued physical inactivity [55].

The result of this study also examined the relationship between occupation and hypertension related behavior. Unemployment and retirement respondents had higher odds of hypertension related behavior than manual workers. Existing studies of the influence of unemployment and retirement on health showed conflicting results [56,57]. Although employment is always thought to have a positive impact on health, some studies reflected that unemployment is not always harmful for health [58]; retirement and unemployment release individuals from occupational stress, at the same time, increased leisure time to give them more opportunities to form health promoting behavior [59].

We also find the impact of SES on hypertension-related knowledge was stable while it was unstable on behavior. The behavior difference between middle school educated and illiterate individuals increased from 2013 to 2016 (*p* < 0.05), and the behavior difference between unemployed individuals and manual workers decreased from 2013 to 2016 (*p* < 0.05), this suggests that we should pay more attention to the role of education in our actions to promote hypertension-related behavior. In addition to the SES factors, other social demographic influencing factors were also assessed in this study. Our findings reported that self-reported hypertension was associated with hypertension related knowledge which is consistent with the results of Xia Li et al. [14]. Respondents who self-reported suffering from hypertension, who were female, older age, divorced, or widowed were more likely to have sufficient behavior. These findings suggest that measures to improve hypertension-related knowledge and behavior should consider the difference in age, gender, marital status, and hypertensive status.

In general, the performance of community-based comprehensive prevention of hypertension was relatively effective in improving hypertension related knowledge while ineffective in correcting behavior from 2013 to 2016. On one hand, we attributed this result to the double influence of urbanization on recently urbanized residents, and on the other we have to reconsider the measures taken by EPHS. Continued health education is essential to enrich knowledge as education is the key-influencing factor, however, EPHS should take more effective measures to promote residents’ ability to turn knowledge into action. In addition, the present study focused on differences in hypertension related knowledge and behavior among those in various SES and other demographic factors. Since the results reflected social inequality, the most important thing is that EPHS should maintain its welfare character as a fundamental policy of the state by paying more attention to recently urbanized residents, especially individuals with lower education and manual workers.

The major limitations of this study should be addressed. Firstly, it is difficult to prove a causal relationship among the factors based on a cross-sectional study. Secondly, the self-reported hypertension data may be lower than the actual ones.

## 5. Conclusions

From 2013 to 2016, hypertension related knowledge improved while behavior had no significant improvement among recently urbanized residents. Despite the improvement, the hypertension related knowledge of residents is still poor, especially in daily salt intake, medication regimens, and some risk factors of hypertension (mental stress, smoking, and lack of exercise). In the context of urbanization, socioeconomic inequality acted on hypertension related knowledge and behavior. The measures devoting to strengthening health education and transforming knowledge into behavior should be taken into account in the recently urbanized residents. At the same time, emphasis should be placed on the individuals with a low level of education and engaged in manual work.

## Figures and Tables

**Table 1:** Demographic characteristics of respondents.

Demographic Characteristics	2013	2016	*p*
	(*n* = 2268)	(*n* = 2601)	
Age (Years)			0.83
18–40	910 (40.1)	1065 (41.0)	
41–64	935 (41.2)	1062 (40.8)	
≥65	423 (18.7)	474 (18.2)	
Gender			0.69
Male	1070 (47.2)	1212 (46.6)	
Female	1198 (52.8)	1389 (53.4)	
Marital status			0.33
Married	1865 (82.2)	2173 (83.5)	
Unmarried	210 (9.3)	236 (9.1)	
Divorced or widowed	193 (8.5)	192 (7.4)	
Education			<0.001
Illiterate	217 (9.6)	205 (7.9)	
Elementary school	674 (29.7)	623 (24.0)	
Middle school	772 (34.0)	770 (29.6)	
Senior school or Technical Secondary School	370 (16.3)	471 (18.1)	
Junior college and above	235 (10.4)	532 (20.5)	
Occupation			<0.001
Manual work	529 (23.3)	577 (22.2)	
Nonmanual work	643 (28.4)	1027 (39.5)	
Unemployment	950 (41.9)	630 (24.2)	
Retirement	146 (6.4)	367 (14.1)	
Monthly income (CNY)			<0.001
<1000	415 (18.3)	63 (2.4)	
1000–2499	1439 (63.4)	1523 (58.5)	
2500–3999	282 (12.4)	652 (25.1)	
≥4000	132 (5.9)	363 (14.0)	
Self-reported hypertension	248 (10.9)	298 (11.5)	0.59

**Table 2:** The correct rate of hypertension related knowledge and behavior questions.

Questions	Correct %		OR *	95% CI	*p*
	2013	2016			
Health knowledge					
1. People should consume no more than 6 g of salt per day	107 (4.7)	194 (7.5)	1.26	0.96–1.65	0.09
2. Patients with hypertension should take medicine for life	859 (37.9)	1099 (42.3)	1.09	0.96–1.24	0.19
3. Obesity is a risk factor for hypertension	594 (26.2)	1443 (55.5)	3.43	3.01–3.92	<0.001
4. Excessive salt is a risk factor for hypertension	701 (30.9)	1615 (62.1)	3.21	2.81–3.67	<0.001
5. Mental stress is a risk factor for hypertension	390 (17.2)	983 (37.8)	2.69	2.32–3.12	<0.001
6. Smoking is a risk factor for hypertension	342 (15.1)	838 (32.2)	2.52	2.16–2.94	<0.001
7. Lack of exercise is a risk factor for hypertension	343 (15.1)	922 (35.4)	1.73	1.37–2.19	<0.001
8. Too much sugar is not a risk factor for hypertension	333 (14.7)	434 (16.7)	0.88	0.74–1.05	0.15
Health behavior					
1. Quit smoking or no smoking	1668 (73.5)	1975 (75.9)	1.1	0.92–1.30	0.30
2. Drinking occasionally or no drinking	2049 (90.3)	2274 (87.4)	0.67	0.54–0.83	<0.001
3. Maintain a low-salt diet	1954 (86.2)	2216 (85.2)	0.90	0.76–1.07	0.24
4. Eat little smoked or pickled food	1814 (80.0)	2244 (86.3)	1.40	1.19–1.66	<0.001
5. Exercise regularly	1211 (53.4)	1706 (65.6)	1.66	1.46–1.89	<0.001
6. Weight control	2185 (96.3)	2434 (93.6)	0.48	0.36–0.64	<0.001
7. Measure blood pressure regularly	1594 (70.3)	701 (27.0)	0.12	0.10–0.14	<0.001

* Adjust for age, gender, marital status, education, occupation, monthly income, and self-reported hypertension.

**Table 3:** Overall score of hypertension related knowledge and behavior.

	Median (IQR)		*p* *
	2013	2016	
Health knowledge	1 (0,3)	3 (1,5)	<0.001
Health behavior	6 (5,6)	5 (5,6)	<0.001

IQR: interquartile range; * Adjust for age, gender, marital status, education, occupation, monthly income, and self-reported hypertension.

**Table 4:** Logistic regression analysis of hypertension related knowledge and behavior with interact effects.

	Hypertension Related Knowledge						Hypertension Related Behavior					
	Model 1			Model 2			Model 1			Model 2		
	OR	95%CI	*p*	OR	95%CI	*p*	OR	95%CI	*p*	OR	95%CI	*p*
**Female**	1.15	0.97–1.37	0.10	1.15	0.96–1.36	0.12	2.99	2.64–3.39	<0.001	2.99	2.64–3.39	<0.001
**Age (Years)**												
41–64	1.09	0.85–1.39	0.51	1.07	0.84–1.37	0.60	1.63	1.37–1.93	<0.001	1.58	1.33–1.88	<0.001
≥65	1.16	0.80–1.67	0.44	1.13	0.78–1.64	0.51	2.76	2.13–3.57	<0.001	2.69	2.07–3.48	<0.001
**Marital status**												
Unmarried	0.77	0.57–1.05	0.09	0.78	0.58–1.05	0.10	0.92	0.74–1.15	0.47	0.92	0.74–1.15	0.47
Divorced or widowed	0.74	0.50–1.09	0.12	0.74	0.50–1.08	0.12	0.75	0.58–0.96	0.02	0.74	0.58–0.96	0.02
**Education**												
Elementary school	1.41	0.93–2.13	0.11	1.99	0.47–8.54	0.35	1.14	0.88–1.45	0.31	0.69	0.32–1.51	0.36
Middle school	2.16	1.41–3.32	<0.001	1.88	0.43–8.22	0.40	1.10	0.84–1.42	0.50	0.44	0.20–0.97	0.04
Senior school or technical secondary school	3.34	2.11–5.30	<0.001	4.67	0.99–21.96	0.05	1.72	1.28–2.31	<0.001	0.78	0.32–1.89	0.58
Junior college and above	5.16	3.16–8.43	<0.001	4.34	0.83–22.65	0.08	2.21	1.59–3.06	<0.001	1.05	0.39–2.82	0.92
**Occupation**												
Nonmanual work	1.07	0.84–1.36	0.59	1.82	0.74–4.44	0.19	1.14	0.96–1.35	0.14	1.53	0.88–2.66	0.13
Unemployment	1.10	0.85–1.43	0.48	1.56	0.62–3.90	0.35	1.44	1.21–1.72	<0.001	2.87	1.64–4.99	<0.001
Retirement	1.22	0.87–1.72	0.25	0.62	0.12–3.05	0.55	1.57	1.22–2.01	<0.001	1.74	0.72–4.23	0.22
**Monthly income (CNY)**												
1000–2499	1.39	0.97–2.01	0.08	1.81	0.59–5.58	0.30	1.18	0.95–1.46	0.13	1.06	0.52–2.18	0.87
2500–3999	1.16	0.77–1.73	0.48	0.76	0.18–3.20	0.71	1.26	0.98–1.61	0.07	1.44	0.61–3.41	0.41
≥4000	1.33	0.86–2.05	0.20	1.49	0.31–7.19	0.62	1.27	0.95–1.68	0.10	2.24	0.80–6.32	0.13
**Self-reported hypertension**	2.34	1.79–3.07	<0.001	2.34	21.78–3.08	<0.001	1.89	1.52–2.35	<0.001	1.90	1.53–2.37	<0.001
**2016 vs. 2013**	1.95	1.61–2.36	<0.001	2.76	0.84–9.13	0.09	0.58	0.51–0.66	<0.001	0.48	0.23–1.02	0.05
**Sufficient knowledge**	–	–	–	–	–	–	1.40	1.17–1.68	<0.001	2.24	1.12–4.48	0.02
**Education × 2016/2013**												
Elementary school				0.80	0.34–1.86	0.61				1.38	0.85–2.24	0.20
Middle school				1.08	0.46–2.53	0.87				1.85	1.13–3.03	0.02
Senior school or technical secondary school				0.80	0.33–1.96	0.63				1.68	0.98–2.89	0.06
Junior college and above				1.09	0.42–2.79	0.87				1.64	0.91–2.97	0.10
**Occupation × 2016/2013**												
Nonmanual work				0.73	0.43–1.22	0.23				0.81	0.58–1.15	0.24
Unemployment				0.80	0.47–1.37	0.42				0.62	0.44–0.88	0.01
Retirement				1.44	0.61–3.37	0.40				0.92	0.56–1.53	0.75
**Monthly income × 2016/2013**												
1000–2499				0.82	0.35–1.91	0.65				1.08	0.60–1.95	0.80
2500–3999				1.19	0.45–3.14	0.72				0.91	0.48–1.74	0.78
≥4000				0.90	0.32–2.53	0.84				0.72	0.35–1.48	0.37
**Sufficient knowledge × 2016/2013**				-	-	-				0.76	0.51–1.12	0.16

OR—odds ratio; CI—confidence interval; the reference group for gender was male, for age was 18–40 years old, for material status was married, for education was illiterate, for occupation was manual work, and for monthly income was <1000.
